# A one-dimensional quantum walk with multiple-rotation on the coin

**DOI:** 10.1038/srep20095

**Published:** 2016-01-29

**Authors:** Peng Xue, Rong Zhang, Hao Qin, Xiang Zhan, Zhihao Bian, Jian Li

**Affiliations:** 1 Department of Physics, Southeast University, Nanjing 211189, China

## Abstract

We introduce and analyze a one-dimensional quantum walk with two time-independent rotations on the coin. We study the influence on the property of quantum walk due to the second rotation on the coin. Based on the asymptotic solution in the long time limit, a ballistic behaviour of this walk is observed. This quantum walk retains the quadratic growth of the variance if the combined operator of the coin rotations is unitary. That confirms no localization exhibits in this walk. This result can be extended to the walk with multiple time-independent rotations on the coin.

Quantum walks (QWs) are valuable in diverse areas of science, such as quantum algorithms^1^,^2^,^3^,^4^,^5^,^6^, quantum computing^7^,^8^,^9^, transport in biological systems^10^,^11^ and quantum simulations of physical system and important phenomena such as Anderson localization^12^,^13^,^14^,^15^,^16^,^17^,^18^,^19^, Bloch oscillation^20^,^21^,^22^,^23^ and non-trivial topological structure^24^,^25^,^26^.

We study one possible route to the localization effect for the QW on the line: the use of multiple-rotation on the coin in order to change interference pattern between paths^27^. We find exact analytical expressions for the time-dependence of the first two moments 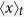 and 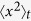, show the behaviour of QWs with two time-independent rotations on the coin and present that a ballistic behaviour instead of localization is observed. This result can be extended to the walk with multiple time-independent rotations on the coin.

## Results

The unitary operator for single-step of this QW with two time-independent rotations on the coin is


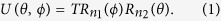


**Figure 1:**
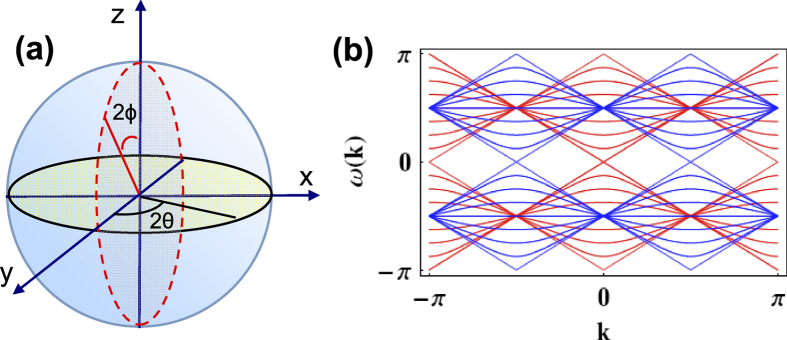
(**a**) Bloch sphere representation of the rotations on the coin. (**b**) Band structure in the first Brillouin zone for rotation parameters 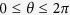 and 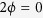 in red lines or 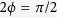 in blue lines. The second rotation along *x* axis allows to close the quasi-energy gap for 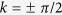.

The two rotations on the coin shown in Fig. 1a


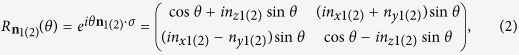


where 
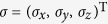
 is the vector of Pauli matrices. The rotations are followed by a conditional position shift operator


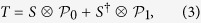


where 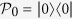 and 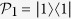 are two orthogonal projectors on the Hilbert space of the coin spanned by 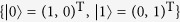, 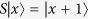 and 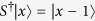 are applied on the walker’s position. One can identify the eigenvectors 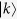 of *S* and 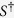,


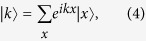


with eigenvalues


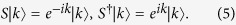


Here a discrete-time QW is considered as a stroboscopic realization of static effective Hamiltonian, defined via the single-step evolution operator


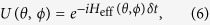


where 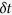 is the time it takes to carry out one step and we set 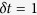 in the followings. The evolution operator for *N* steps is given by 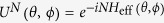. For the general rotations in Eq. (2), the effective Hamiltonian can be written as


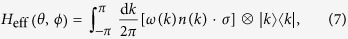


where the quasi-energy (Fig. 1b)


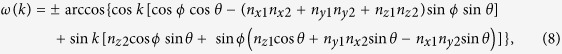


and the unit vector 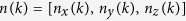



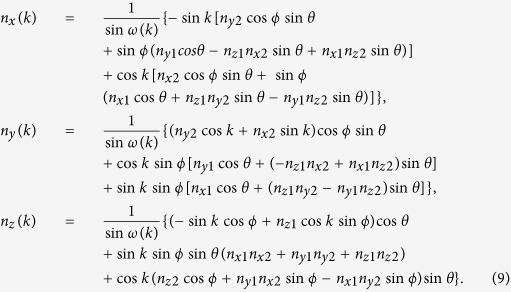


The inverse Fourier transformation is 
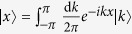
. The initial state of the walker + coin system can be written as 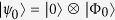, where the original position state of the walker is 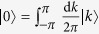. In the *k* basis, the evolution operator 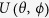 becomes


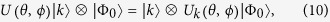


where


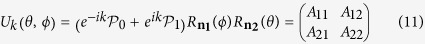


is a 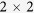 unitary matrix with the matrix elements


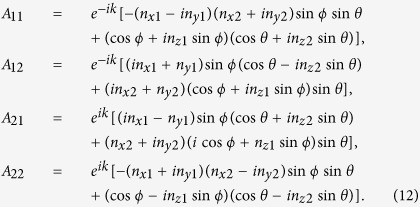


At time *t* (the time *t* is proportional to the step number *N*), the walker + coin state evolves to


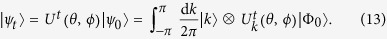


The probability for the walker to reach a position *x* at time *t* is


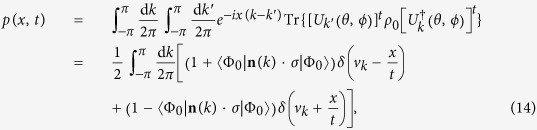


where 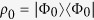, the group velocity of the walker 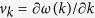. To determine if there is localization effect, we care more about the position variance and the dependence of the variance on time. Thus we restrict our interest to the moments of the distribution


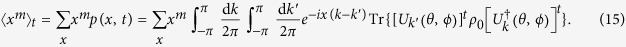


With the formula of the delta function 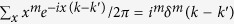, the expression of the *m*th moment is rewritten as


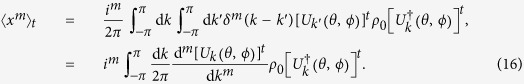


Similar to a Hadamard coined walk^28^, one can find the eigenvectors 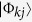 of 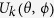 and corresponding eigenvalues 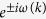. We can expand the initial coin state 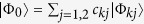. With 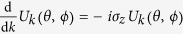
29, we only keep the diagonal non-oscillatory terms and obtain


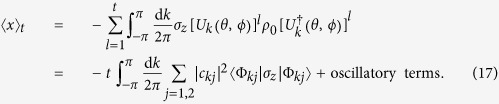


For non-degenerate unitary matrix 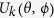, except for the diagonal non-oscillatory terms, most of the terms are oscillatory, which average to zero in the long-time limit^29^.

Similarly, the second moment is obtained


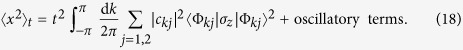


From Eqs. (8) and (11), we can see the spectrum 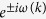 of 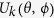 is non-degenerate. Even for degenerate 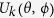 one can modify Eqs. (17) and (18) to include appropriate cross terms, which does not change the dependence of the position variance on time.

Generically, in the long-time limit, for a unitary coin the first moment of the QW undergoes a linear drift and the variance grows quadratically with time. There is a special case—the 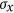 coined QW, i.e., 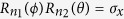, in which the eigenstates of 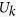 are 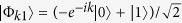 and 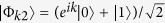, resulting in 
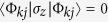
 (for 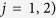. Thus the variance of the 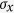 coined QW does not depend on time.

In the two rotations case, the combination operation of two rotations on the coin 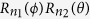 shown in Eq. (11) is unitary. Thus for arbitrary choices of parameters *θ* and 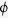 the position variance of the QW with two time-independent rotations on the coin grows quadratically and the behaviour of the QW is ballistic. Therefore, a second coin rotation does not change the behaviour of QW from a ballistic spread to localization.

The asymptotic analysis of the behaviour of this QW with two time-independent coin rotations can be extended to more general QW with more time-independent rotations on the coin. Once the combined operator of the multiple-rotation on the coin is unitary, the position variance grows quadratically with time and this QW shows ballistic behaviour. No localization effect occurs.

This walk is homogeneous in either spatial or temporal space. The coin rotations do not cause inhomogeneity in this walk which usually leads to interesting localization effect.

## Discussion

In summary, we study the QW with two time-independent rotations on the coin through the analytical solutions for the time dependence of the position variance. The asymptotic result can be extended to the walk with multiple time-independent rotations on the coin. As long as the combination of the multi-rotations is unitary, the variance grows quadratically with time and the QW shows ballistic behaviour. No localization effect is observed in this QW. Although the fact that two topics—QWs and localization effect meet, is fascinating and opens the door to rich theoretical and experimental investigation of quantum phenomena. Thus not only the investigation on simulating localization with QWs but also the study on the limitations on localization in quantum walk are important and worthy of attention. Our research exactly gives insight into limitations on localization.

## Additional Information

**How to cite this article**: Xue, P. *et al.* A one-dimensional quantum walk with multiple-rotation on the coin. *Sci. Rep.* **6**, 20095; doi: 10.1038/srep20095 (2016).
